# Comparison of Mitochondrial Reactive Oxygen Species Production of Ectothermic and Endothermic Fish Muscle

**DOI:** 10.3389/fphys.2017.00704

**Published:** 2017-09-15

**Authors:** Lilian Wiens, Sheena Banh, Emianka Sotiri, Martin Jastroch, Barbara A. Block, Martin D. Brand, Jason R. Treberg

**Affiliations:** ^1^Department of Biological Sciences, University of Manitoba Winnipeg, MB, Canada; ^2^Helmholtz Diabetes Center at Helmholtz Zentrum München, Institute for Diabetes and Obesity Munich, Germany; ^3^Tuna Research and Conservation Center, Hopkins Marine Station, Stanford University Stanford, CA, United States; ^4^Buck Institute for Research on Aging Novato, CA, United States; ^5^Department of Human Nutritional Sciences, University of Manitoba Winnipeg, MB, Canada

**Keywords:** endothermy, ectothermy, tuna, mitochondrial energetics, superoxide, hydrogen peroxide, fractional electron leak

## Abstract

Recently we demonstrated that the capacity of isolated muscle mitochondria to produce reactive oxygen species, measured as H_2_O_2_ efflux, is temperature-sensitive in isolated muscle mitochondria of ectothermic fish and the rat, a representative endothermic mammal. However, at physiological temperatures (15° and 37°C for the fish and rat, respectively), the fraction of total mitochondrial electron flux that generated H_2_O_2_, the fractional electron leak (FEL), was far lower in the rat than in fish. Those results suggested that the elevated body temperatures associated with endothermy may lead to a compensatory decrease in mitochondrial ROS production relative to respiratory capacity. To test this hypothesis we compare slow twitch (red) muscle mitochondria from the endothermic Pacific bluefin tuna (*Thunnus orientalis*) with mitochondria from three ectothermic fishes [rainbow trout (*Oncorhynchus mykiss*), common carp (*Cyprinus carpio*), and the lake sturgeon (*Acipenser fulvescens*)] and the rat. At a common assay temperature (25°C) rates of mitochondrial respiration and H_2_O_2_ efflux were similar in tuna and the other fishes. The thermal sensitivity of fish mitochondria was similar irrespective of ectothermy or endothermy. Comparing tuna to the rat at a common temperature, respiration rates were similar, or lower depending on mitochondrial substrates. FEL was not different across fish species at a common assay temperature (25°C) but was markedly higher in fishes than in rat. Overall, endothermy and warming of Pacific Bluefin tuna red muscle may increase the potential for ROS production by muscle mitochondria but the evolution of endothermy in this species is not necessarily associated with a compensatory reduction of ROS production relative to the respiratory capacity of mitochondria.

## Introduction

Fishes of the family Scombridae (the mackerels, bonitos, and tunas) are highly active marine predators. Within this clade, endothermy has evolved and is most expressed in the three species of bluefin tunas that occupy cooler temperate waters and, as adults, subpolar seas (Block et al., 2005; Boustany et al., 2007; Whitlock et al., 2015). Endothermy is also found in members of the tribe Thunnini (the tunas), and other fishes including cranial endothermy in billfishes and butterfly mackerel, endothermy in lamnid sharks, and systemic endothermy in the Opah (Wegner et al., 2015). Since temperature can affect the rate of biological processes, including enzymatic reaction rates, such endothermy has been a particular focus for comparative physiologists. The research on heat production, evolutionary convergence, and potential selective advantages of endothermy in fishes have been discussed and reviewed elsewhere (for examples see Block, 1994; Block and Finnerty, 1994; Bernal et al., 2001; Dickson and Graham, 2004; Graham and Dickson, 2004).

Bluefin tunas maintain elevated slow-twitch red muscle temperatures in the core of the body, up to 21°C above surrounding ambient water temperatures (Stevens et al., 2000; Marcinek et al., 2001; Blank et al., 2007), and swim constantly to respire. Mitochondria must play a key role in resupplying the ATP that is constantly being consumed by muscle contraction. Although, mitochondria are the primary consumers of oxygen during aerobic metabolism they can also produce reactive oxygen species (ROS), primarily as superoxide and H_2_O_2_. ROS production is generally measured as H_2_O_2_ efflux, which combines the net production of superoxide and H_2_O_2_. Excessive production of ROS may lead to macromolecular damage (Murphy, 2009; Jastroch et al., 2010). Studies on mitochondria isolated from ectotherms indicate that the rate of ROS production increases with temperature (Abele et al., 2002; Heise et al., 2003; Chung and Schulte, 2015; Banh et al., 2016); raising the possibility that the warming of specific regions in endothermic fish may also result in increased potential for mitochondrial H_2_O_2_ efflux. If higher physiological temperatures could lead to elevated potential for ROS production then compensatory mechanisms to minimize mitochondrial H_2_O_2_ production may co-evolve with endothermy.

We recently demonstrated that H_2_O_2_ efflux from muscle mitochondria isolated from three ectothermic fishes was much lower than rates for rat muscle mitochondria when assayed at physiological temperatures of 15° and 37°C for the ectothermic fish and rat respectively (Banh et al., 2016). However, the fractional electron leak (FEL), a value that normalizes ROS production relative to total mitochondrial electron flux, was markedly higher for fish mitochondria compared to the rat at physiological temperatures (Banh et al., 2016). The high FEL in the fish suggests that relative to the overall metabolic capacity of the mitochondria (including the electron transport chain and Krebs cycle where the majority of superoxide/H_2_O_2_ producing complexes are found) ectothermic fish mitochondria have intrinsically higher potential for H_2_O_2_ production compared to the rat. For all species examined the rate of mitochondrial H_2_O_2_ production was greater at higher temperatures. Indeed, in some cases, mitochondria from trout and carp produced as much or more H_2_O_2_ at 25°C than rat mitochondria did at 37°C (Banh et al., 2016). Taken together these findings suggest that muscle mitochondria from ectotherms may have a greater propensity for H_2_O_2_ production compared to a representative endotherm.

Our recent work proposed that mitochondrial H_2_O_2_ producing and consuming pathways have differing temperature sensitivities (Banh et al., 2016). This thermal mismatch hypothesis of mitochondrial H_2_O_2_ metabolism (Banh et al., 2016) predicts that compensatory mechanisms may have been crucial in the evolution of endothermy because excess ROS production would be associated with oxidative stress and disruption of redox homeostasis. Thus, one can hypothesize that some compensatory response to mitigate the influence of elevated body temperatures could be concomitant with the evolution of endothermy. The current study tests this hypothesis by comparing mitochondria isolated from red muscle of the endothermic Pacific Bluefin tuna (*Thunnus orientalis*), maintained at an elevated 25°C, with three ectothermic fishes at 15°C. For reference we also evaluate the well-studied rat as a representative endothermic mammal. For all species the respiratory capacity and rate of H_2_O_2_ formation was measured at the respective physiological temperatures and a common temperature of 25°C to test if tuna had reduced potential for ROS formation relative to their mitochondrial metabolic capacity (estimated by respiration rate). Overall, we find that tuna red muscle mitochondria are similar to those of ectothermic fish species in both the capacity and thermal sensitivity of ROS formation. Thus, enhanced mitochondrial superoxide/H_2_O_2_ production may be an underappreciated consequence of the tissue warming found in the muscle of endothermic fishes.

## Materials and methods

### Animals and sampling

All animal use procedures were based on the policies from the Canadian Council on Animal Care and approved by the University of Manitoba Fort Garry Campus Animal Care Committee (for ectothermic fish and rat) or the Stanford Administrative Panel on Laboratory Animal Care Committee in accordance to all policies of Stanford University (tuna).

#### Pacific bluefin tuna

Pacific bluefin tuna (*T. orientalis*) were housed at the Tuna Research and Conservation Center at Stanford University's Hopkins Marine Station, Pacific Grove, CA. Animal collection, transport and husbandry were the same as previously reported (Galli et al., 2009; Clark et al., 2010) and all animals in this study were acclimated to 20°C prior to experimental initiation for at least 30 days. Fish were of both sexes and ~13–15 kg and 80–90 cm in fork length. For sampling, fish were killed by pithing, followed by spinal severance at the junction between the spine and skull. Tissues were immediately removed and placed in ice-cold isolation medium for preparation of mitochondria.

#### Ectothermic fish

The ectothermic fishes in this study include two other teleosts, the relatively high aerobic capacity rainbow trout (*Oncorhynchus mykiss*) and the low activity benthic-dwelling common carp (*Cyprinus carpio*) as well as the lake sturgeon (*Acipenser fulvescens*), a relatively inactive Acipenseriform species. Rainbow trout and carp were obtained from commercial suppliers. Lake sturgeon were reared from eggs fertilized at the University of Manitoba. All ectothermic fish were maintained at 15°C, for at least 8 weeks, on a 12:12 photoperiod in flow-through tanks and fed commercial pelleted food to satiation at least every other day. Fish were of both sexes and were ~1.5–4.5 kg. For sampling, fish were killed by a blow to the head, followed by severing of the gill arches, and tissues were immediately dissected out and placed in ice-cold isolation medium.

#### Rats

Male Sprague–Dawley rats, ~250–400 g, were from the University of Manitoba and housed at the Duff–Roblin Animal Holding facility in standard cages with wood shaving or cardboard bedding along with shelters, washers and other plastic environmental enrichment. Rats had *ad libitum* access to chow and water. Rats were killed by asphyxiation with *CO*_2_ followed by pneumothorax and hindlimb muscle was dissected off and put in ice-cold isolation medium prior to processing for isolation of mitochondria.

### Mitochondrial isolation

Tissues were rinsed in ice-cold isolation medium and diced to facilitate homogenization. Two isolation media were used in this study. For tuna, we used 140 mM KCl, 20 mM HEPES, 2 mM EGTA, pH 7.0 at 20°C whereas 120 mM KCl, 20 mM HEPES, 1 mM EGTA, pH 7.1 at 20°C was used for the rats and carp. For rainbow trout and sturgeon we tested both isolation media and found no difference in results. All steps were maintained at 0–4°C. The procedure for rats (Affourtit et al., 2012) and the ectothermic fishes (Banh et al., 2016) are described in detail elsewhere but, briefly, involved incubating diced tissue with a protease followed by disruption by homogenization and isolation of mitochondria by differential centrifugation. The final pellet was resuspended in a small volume of isolation medium at ~20–45 mg mitochondrial protein · ml^−1^ (determined by biuret assay with BSA as standard).

For tuna, diced tissue was mixed with ice-cold homogenization medium (isolation medium plus 0.5% w/v BSA) and homogenized with a motor-driven Teflon (Polytetrafluoroethylene) to glass homogenizer. The homogenate was centrifuged for 5 min at 900 × *g*. The supernatant was collected and filtered through a fine plastic mesh to aid in lipid and particulate removal. Following filtration, the supernatant was centrifuged at 9,000 × *g* for 10 min. The resulting pellet was washed by resuspending in isolation medium followed by centrifugation 9,000 × *g* for 10 min. Care was taken to not transfer any of the lipid in the supernatant or resuspend any material that was clearly not mitochondria (for example red blood cells and particulate matter). The resulting pellet was washed a second time (as described above) and the final pellet was resuspended in a small volume of isolation medium at ~30–60 mg mitochondrial protein · ml^−1^ (determined by biuret assay with BSA as standard).

### Mitochondrial H_2_O_2_ production

Combined superoxide and hydrogen peroxide production by mitochondria was measured spectrofluorometrically as H_2_O_2_ efflux in the same medium as used for respiration. In some cases phosphate was omitted or 1 μg · ml^−1^ oligomycin was added, we found these had relatively little (c. 10% or less) influence on the observed rates and thus pooled all data. The assay medium also contained an H_2_O_2_ detection system consisting of 5 IU · ml^−1^ of horseradish peroxidase, 25 IU · ml^−1^ superoxide dismutase, and 50 μM Amplex UltraRed in a total volume of 2 ml using either an Agilent Eclipse or a Shimadzu RF-5301PC. Fluorescence was monitored, with constant stirring, at 560 nm excitation and 590 nm emission wavelengths, respectively. Raw fluorescence values were transformed to moles of H_2_O_2_ based on calibration with known amounts of H_2_O_2_. Background rates of product formation prior to the addition of substrate were low (typically < 10% of rate with substrate) and were subtracted from rates in the presence of substrate.

### Mitochondrial respiration

Mitochondrial substrate oxidation was measured in a water-jacketed chamber with a Rank Brothers Clark-type oxygen electrode (Cambridge, U.K) or using the Oroboros O2K (Innsbruck, Austria). Tuna mitochondria used the following respiration medium: 140 mM KCl, 20 mM Hepes, 1 mM EGTA, 5 mM K_2_HPO_4_, and 0.5% (w/v) BSA, pH 7.3 at 20°C. For ectotherm fish and rats the standard medium was 120 mM KCl, 20 mM Hepes, 1 mM EGTA, 5 mM K_2_HPO_4_, and 0.3% (w/v) BSA, pH 7.4 at 20°C. Note, we found comparable (within 10%) results between the two respiration media with the ectothermic fish mitochondria.

Following addition of mitochondria to the chamber the rates of oxygen consumption were measured under the following respiratory conditions: (i) non-phosphorylating, in the presence of exogenous substrate (state 2); (ii) phosphorylating, the rate with exogenous substrate plus 500 or 800 μM ADP (state 3); (iii) a pharmacologically induced non-phosphorylating state, with 1 μg · ml^−1^ oligomycin (which blocks ATP synthesis) (state 4o). These conditions were measured in sequence and the respiratory control ratios (RCR) were calculated as the rate of oxygen consumption of mitochondria in state 3/state 4o.

### Data analysis

All data are shown as mean ± SEM. Values were compared either by ANOVA (with Student-Newman–Keuls *post-hoc* test) or *t*-test (Welch's) with *p* < 0.05 being considered significant.

To determine the FEL we calculated values based on:

$$\begin{array}{l} \text{FEL}=100\times [(\text{rate of }{\text{H}}_{\text{2}}{\text{O}}_{\text{2}}\text{ efflux})\times (\text{rate of }{\text{H}}_{\text{2}}{\text{O}}_{\text{2}}\text{ efflux}\\ \;{+\text{ rate of }{\text{O}}_{\text{2}}\text{ consumption})}^{-1}] \end{array}$$

Where H_2_O_2_ efflux rate is in nmol min^−1^ mg protein^−1^ and O_2_ consumption rate is in nmol O min^−1^ mg protein^−1^. In some cases rates of H_2_O_2_ efflux and O_2_ consumption could not be measured at all assay temperatures for mitochondria isolated from the same individual animal therefore values are expressed as mean ± SEM where the SEM was derived by standard error propagation procedures. Differences among these mean values were compared by 2-tailed Welch's *t*-test (*p* < 0.05 being considered significant).

## Results

### Interspecific patterns in rates of H_2_O_2_ production

**Figure 1:**
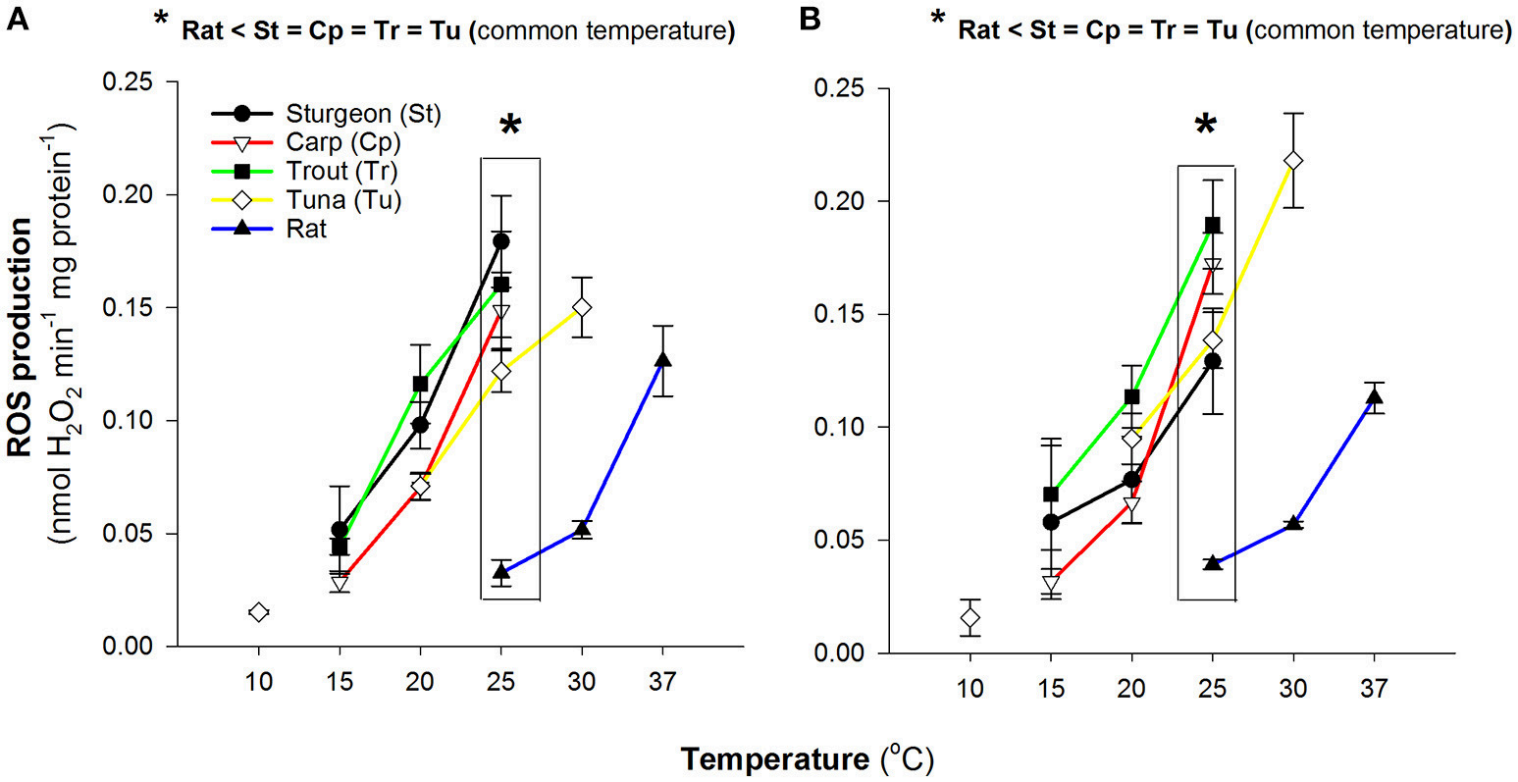
Comparison of reactive oxygen species production by isolated muscle mitochondria. Data are mean ± SEM (*n* = 3–5 depending on species) and substrates were 5 mM pyruvate and malate **(A)** or 0.05 mM D/L-palmitoylcarnitine and 5 mM malate **(B)**. Assay temperature is indicated and rates were determined as H_2_O_2_ efflux normalized to mitochondrial protein. Physiologically relevant temperatures are 15°C for sturgeon, carp and trout; 25°C for Bluefin tuna; 37°C for rat.

Aerobic locomotion in both fish and mammals is largely fuelled by carbohydrate and lipid oxidation (Lauff and Wood, 1996, 1997; McClelland, 2004), as such, we evaluated the potential for mitochondrial ROS formation during the oxidation of either pyruvate or palmitoylcarnitine to reflect these respective fuels. In both cases saturating malate was also included to alleviate the trapping of intramitochondrial coenzyme-A as acetyl-CoA by the provisioning of oxaloacetate and to supply NADH for complex I via malate dehydrogenase. When compared at physiological temperatures, 15°, 25°, and 37°C for ectothermic fish, Bluefin tuna and rat respectively, there was a pattern of increasing capacity for ROS production as physiological temperature increased but the tuna mitochondrial ROS production at 25°C was comparable to the rat mitochondria at 37°C (Figure 1).

In all species the mitochondrial H_2_O_2_ production was sensitive to temperature (Figure 1) with similar sensitivity across species. At higher temperatures the rate of H_2_O_2_ production was increased. Overall the rates of H_2_O_2_ production by isolated Pacific bluefin tuna mitochondria were comparable to other fishes. When measured at a common assay temperature of 25°C, the physiological temperature for the bluefin tuna red muscle, rates of H_2_O_2_ production by tuna mitochondria were not different from mitochondria isolated from the other fishes (Figure 1). In all cases the rates at 25°C in fish mitochondria were markedly higher than the rate seen with rat muscle mitochondria with both carbohydrate and lipid based oxidative substrates (Figure 1). Within species we found no pattern of difference in rates of H_2_O_2_ formation between oxidation of pyruvate or palmitoylcarnitine (Figures 1A,B).

### Comparisons of respiratory rates

**Figure 2:**
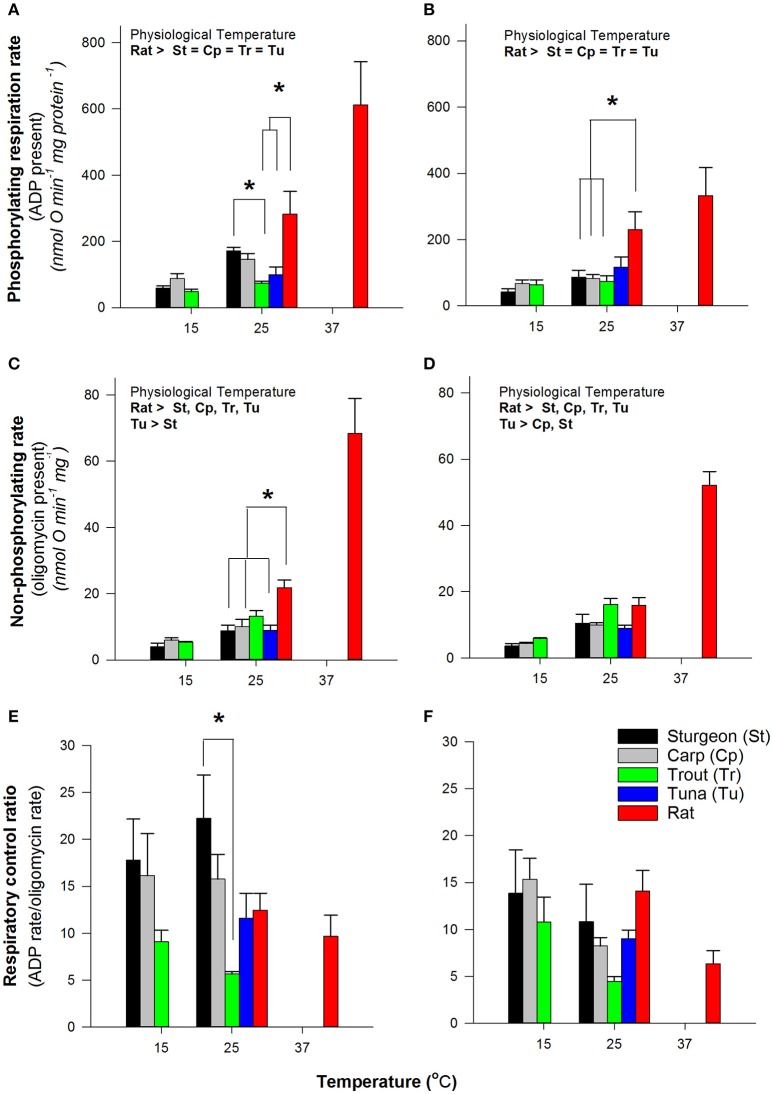
Comparison of respiration rates by isolated muscle mitochondria. Data are mean ± SEM (*n* = 3–5 depending on species) and substrates were 5 mM pyruvate and malate **(A,C,E)** or 0.05 mM D/L-palmitoylcarnitine and 5 mM malate **(B,D,F)**. Assay temperature is indicated and contrasts were made at a common temperature (indicated by ^*^ and brackets) or physiological temperatures as indicated within figures. Physiologically relevent temperatures are 15°C for sturgeon, carp and trout; 25°C for Bluefin tuna; 37°C for rat.

To evaluate if the differences seen in H_2_O_2_ production rates might be linked to mitochondrial respiratory capacity we measured the rates of respiration at physiological temperatures as well as at a common temperature of 25°C. Under conditions of phosphorylating respiration (presence of ADP) and non-phosphorylating respiration (absence of ADP phosphorylation) there was an overall tendency for increasing rates with increasing physiological temperature (Figure 2). At the common temperature of 25°C the rates of respiration with either pyruvate or palmitoylcarnitine (both supplemented with malate) were not different across fish mitochondria, with the exception of sturgeon being higher than trout with pyruvate and malate under phosphorylating conditions (Figure 2). Rates of respiration were generally higher for rat mitochondria. The respiratory control ratio (RCR) was not affected by assay temperature except for trout (Figure 2C) and at 25°C the RCR for Pacific bluefin tuna was not different from the other fishes. The stability of RCR values across temperatures suggests collapsing mitochondrial coupling efficiency with changing temperature is an unlikely explanation for differences in H_2_O_2_ production with assay temperature.

### Fractional electron leak

**Figure 3:**
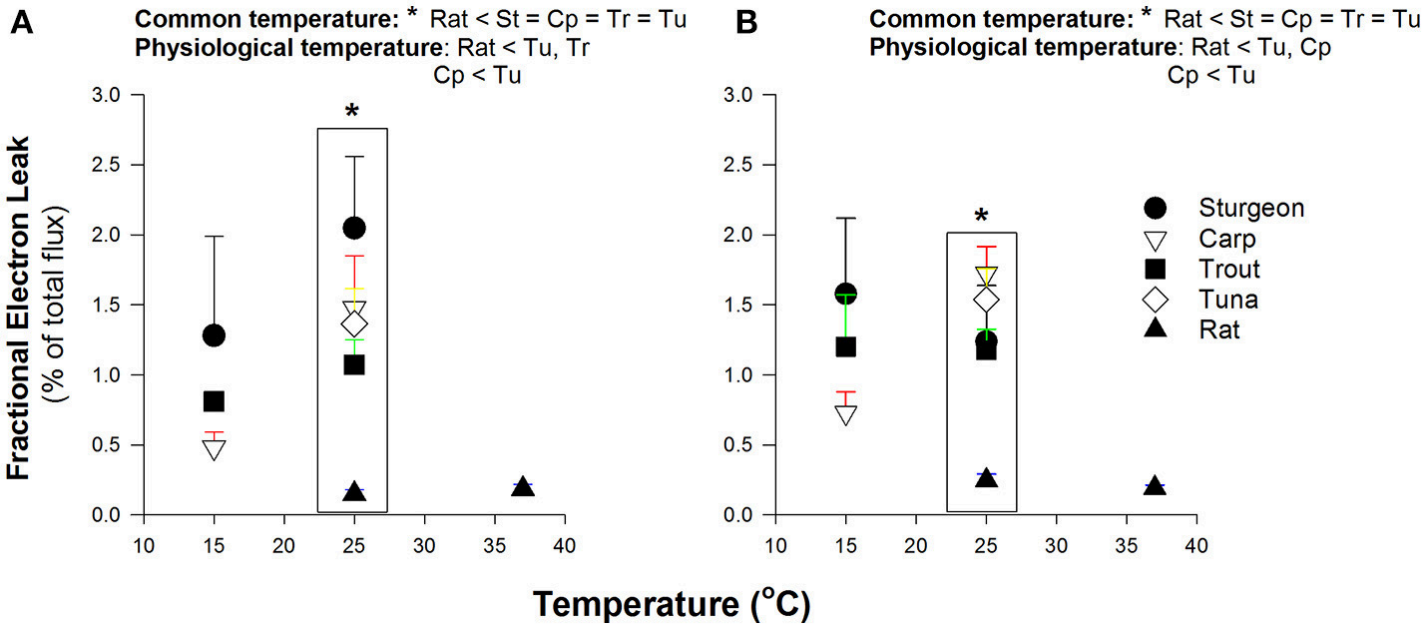
Fractional electron leak by isolated muscle mitochondria. Data are mean ± SEM (*n* = 3–5 depending on species) and substrates were 5 mM pyruvate and malate **(A)** or 0.05 mM D/L-palmitoylcarnitine and 5 mM malate **(B)**. Assay temperature is indicated and contrasts were made at a common temperature (indicated by ^*^ and brackets) or physiological temperatures as indicated within figures. Physiologically relevent temperatures are 15°C for sturgeon, carp and trout; 25°C for Pacific bluefin tuna; 37°C for rat. Species abbreviations are as indicated in Figures 1, 2.

At a common assay temperature of 25°C the FEL for all fish species was markedly greater than for rat, with Pacific bluefin tuna being unexceptional in comparison to the other fish (Figure 3). For species where two test temperatures were used (all species but the tuna) there was a tendency for increasing FEL with higher assay temperature. This thermal sensitivity led to some ectothermic fishes having values for FEL that were not different from the rat at physiological temperatures (Figure 3), but the high levels of variation warrant some caution with interpretation of this result.

## Discussion

### Comparisons among fishes

For all species examined the production of H_2_O_2_ by isolated muscle mitochondria was a function of assay temperature. Higher assay temperatures led to higher rates of production (Figure 1). However, across assay temperatures and using all substrate mixes tested, the rates of H_2_O_2_ production by isolated Pacific bluefin tuna mitochondria compared favorably with other fish mitochondria. Similarly, Pacific bluefin tuna mitochondria had similar respiration rates to most other fish red muscle mitochondria when measured at a common assay temperature irrespective of the physiological body temperature for the mitochondria (Figure 2). Our respiration rates with pyruvate also compare well with those for other teleosts over a range of both *in vitro* assay and physiological temperatures (Johnston et al., 1994, 1998) suggesting our mitochondrial preparations are reasonably consistent with those used in other studies on temperature effects on fish mitochondria.

A potential way to separate mitochondrial H_2_O_2_ production from the mitochondrial roles in oxygen consumption is to express the electron leakage to H_2_O_2_ as a fraction of the overall electron flux (FEL). Using the FEL we find no evidence for reduced potential for H_2_O_2_ formation relative to substrate oxidation rates; tuna mitochondria were similar to mitochondria from ectothermic fishes (Figure 3). The respiratory capacity, measured as ADP-stimulated respiration rate, was similar in Pacific bluefin tuna mitochondria to most other fish red muscle mitochondria. This similarity in respiration rate is consistent with no compensatory change in the endothermic tuna red muscle in the overall metabolic systems responsible for electron flux to oxygen either as respiration or leaking as mitochondrial H_2_O_2_ production. Of note, one could argue that the ADP-stimulated rate of oxygen consumption better reflects the overall amount of biochemical “machinery” in the mitochondrion, and thus the phosphorylating rate of respiration (state 3) is a better denominator to normalize H_2_O_2_ production; however, we find the same overall pattern is seen as that for FEL indicating that regardless of the respiration rate used to normalize H_2_O_2_ production all fish muscle mitochondria appear comparable at the same assay temperature (data not shown).

### Comparison of muscle mitochondria from fish and the rat

We included one representative mammal, the rat. Overall, mitochondria from all fish assayed appear to have greater potential for electron leak relative to their respiratory capacity (FEL) compared to the rat. The similarity of FEL across fishes in this study suggests fishes, and possibly other ectotherms, may have a propensity toward increased electron leakage compared to more derived endothermic species. This may represent an adaption to minimize the ROS induced damage that could occur at higher endogenous temperatures common in mammals and birds. This pattern warrants further investigation to determine if it holds more broadly across taxa. Contrasting endothermic and ectothermic representative birds and reptiles would be a particularly interesting comparison along these lines.

### Fuel selection: is there a trade-off between H_2_O_2_ production and specific carbon source?

During aerobic locomotion both lipid and carbohydrate are important carbon sources for oxidation in teleost fishes (Lauff and Wood, 1996, 1997) and mammals (McClelland, 2004). This is consistent with relatively similar rates of pyruvate and fatty acid oxidation by isolated muscle mitochondria from teleosts as seen by us and others (Moyes et al., 1989, 1992; Chamberlin et al., 1991). Thus, pyruvate and palmitoylcarnitine were used as representative carbohydrate- and lipid-based oxidative substrates in the present study.

These physiologically important substrates were also chosen because they are known to produce H_2_O_2_ in isolated mitochondria (reviewed in Brand, 2010). Pyruvate and palmitoylcarnitine may contribute directly to mitochondrial ROS production via the matrix enzymes involved with their metabolism, as well as indirectly by producing NADH and QH_2_ which reduce electron transport chain complexes I and III respectively. It has been reported that palmitoylcarnitine driven respiration in rodent mitochondria may have high capacity for H_2_O_2_ production compared to other NADH-generating substrates (St Pierre et al., 2002; Seifert et al., 2010); although more recent work found the rates comparable to that found with other NADH-generating substrates (Perevoshchikova et al., 2013). If lipid fuelled respiration was to lead to greater potential for H_2_O_2_ production then there is a possibility that the enhanced lipid oxidation proposed for high-performance fishes (Weber and Haman, 1996) may have consequences on mitochondrial H_2_O_2_ production. However, we broadly find comparable rates of H_2_O_2_ production, FEL, and ADP-stimulated respiration rates with isolated muscle mitochondria from several fishes and one mammal. As such, our evidence does not support a trade-off between selection for lipid or carbohydrate oxidation in muscle and potential for H_2_O_2_ production.

### Limitations of the study

#### ADP availability in fish red muscle

The rate of H_2_O_2_ production from mitochondria isolated from several tissues, including brain, liver and muscle, typically declines in the presence of ADP (Starkov and Fiskum, 2003; Starkov et al., 2004; Santiago et al., 2008; Quinlan et al., 2012; Goncalves et al., 2015). This decline appears to be primarily due to decreased protonmotive force and the consequent decreased reduction state of electron carriers and the enzyme complexes involved with electron transport and substrate oxidation. Although, some ADP is always being produced in skeletal muscle due to ATP hydrolysis, we are unaware of precise data to estimate the exact ADP availability typical in the red muscle of the various fish species we examined here. For this reason we use the rate of H_2_O_2_ production in the absence of ADP as a worst case scenario of ROS production under conditions of saturating substrate and oxygen availability. Even though the ADP availability in red muscle of constantly swimming fish should alleviate some ROS formation, our results suggest that mitochondria in a teleost with elevated muscle temperature should be exposed to a greater H_2_O_2_ production rate than if the same mitochondria were respiring at a lower temperature.

#### Underestimations of H_2_O_2_ production due to matrix consumers

Using H_2_O_2_ efflux as a measure of H_2_O_2_ production by isolated mitochondria underestimates the actual production and with rat muscle mitochondria this underestimate is a function of the production rate, with high rates leading to lower underestimates (Munro et al., 2016). It was not possible to undertake the complex procedure to compromise the matrix H_2_O_2_ consuming pathways (see Munro et al., 2016 for full details) and thus we cannot exclude the possibility that patterns seen here may not fully reflect the actual rates of production for each species. As such, it is possible a compensatory increase in antioxidant capacity is found in Bluefin tuna that our dataset may not capture. Alternatively, the increased antioxidant capacity with increasing temperature, even if incomplete as proposed elsewhere (Banh et al., 2016), may be sufficient to compensate for increase H_2_O_2_ production capacity.

#### Size scaling effects

The production of H_2_O_2_ by isolated mitochondria may scale with body size with larger animals having lower rates of H_2_O_2_ production than smaller species (Lambert et al., 2007). We have not tried to account for mass-scaling effects but in the case of the rat, the smallest species used, it would be expected this pattern would disproportionately elevate H_2_O_2_ production. Conversely, rat has the lowest rate of H_2_O_2_ formation at a common assay temperature. For the fishes, the Pacific bluefin tuna was the largest of the species and thus may have somewhat lower rates of H_2_O_2_ production due to scaling effects. Even if the bluefin tuna H_2_O_2_ values are low due to their larger size this would not change our primary thesis in this study: there is no support for reduced H_2_O_2_ production by isolated muscle mitochondria from this endothermic fish species since correcting for mass would be expected to increase the rates in tuna relative to smaller fish species.

#### Muscle fiber type

Finally, all fish muscle mitochondrial preparations were from red muscle, which is almost exclusively slow-twitch muscle because of the anatomical separation of slow and fast twitch fibers in most fishes making fish excellent models for studying muscle mitochondrial function from specific fiber types (Leary et al., 2003). However, the rat muscle preparation will be a mix of fiber types and it has been reported that the H_2_O_2_ formation varies by fiber type in rodent muscle (Anderson and Neufer, 2006). But again the pattern found should have biased the rat mitochondrial preparation toward elevated H_2_O_2_ production because fast-twitch muscle appears to produce more H_2_O_2_ than slow-twitch fibers (Anderson and Neufer, 2006). Therefore, we conclude that our finding of markedly lower H_2_O_2_ formation in the representative mammal should not be compromised but the use of a mixed muscle type for this species alone.

### Summary

The elevated red muscle temperature in endothermic fishes should increase the potential for H_2_O_2_ formation but we see no evidence for compensatory decrease in H_2_O_2_ production in the Pacific Bluefin tuna, whereas we do see this in a representative endothermic mammal. Slow-twitch red muscle has a primary function in locomotion in these fish. This suggests that there is a trade-off in red, slow-twitch muscle between thermal effects that are selected for to improve muscle performance at elevated temperature (Altringham and Block, 1997), and the propensity toward mitochondrial H_2_O_2_ formation in the evolution of endothermy in fishes.

## Author contributions

LW: Helped write initial manuscript draft, contributed original data and data analysis; SB: Contributed original data, analysis and editorial contribution to manuscript; ES: Contributed original data and editorial contribution to manuscript; MJ: Contributed to study design, provided original data, and assisted in writing manuscript; BB: Contributed to study design and with writing of manuscript; MB: Contributed to study design and with writing of manuscript; JT contributed to study design, provided original data, and assisted in writing manuscript.

### Conflict of interest statement

The authors declare that the research was conducted in the absence of any commercial or financial relationships that could be construed as a potential conflict of interest.
